# Shifting cancer care towards Multidisciplinarity: the cancer center certification program of the German cancer society

**DOI:** 10.1186/s12885-017-3824-1

**Published:** 2017-12-14

**Authors:** Christoph Kowalski, Ullrich Graeven, Christof von Kalle, Hauke Lang, Matthias W. Beckmann, Jens-Uwe Blohmer, Martin Burchardt, Michael Ehrenfeld, Jan Fichtner, Stephan Grabbe, Hans Hoffmann, Heinrich Iro, Stefan Post, Anton Scharl, Uwe Schlegel, Thomas Seufferlein, Walter Stummer, Dieter Ukena, Julia Ferencz, Simone Wesselmann

**Affiliations:** 1 German Cancer Society, Department for Certification, Kuno-Fischer-Strasse 8, 14057 Berlin, Germany; 2 Kliniken Maria Hilf GmbH, Viersener Strasse 450, 41063 Mönchengladbach, Germany; 3 NCT University Hospital, Im Neuenheimer Feld 460, 69120 Heidelberg, Germany; 4 University Hospital, Langenbeckstrasse 1, 55131 Mainz, Germany; 5 University Hospital, Universitätsstrasse 21-23, 91054 Erlangen, Germany; 6 Charité University Hospital, Charitéplatz 1, 10117 Berlin, Germany; 7 University Hospital, Fleischmannstrasse 42, 17489 Greifswald, Germany; 8 University Hospital, Lindwurmstrasse 2a, 80337 Munich, Germany; 9 Johanniter Krankenhaus, Steinbrinkstr. 96, 46145 Oberhausen, Germany; 10 University Hospital, Im Neuenheimer Feld 460, 69120 Heidelberg, Germany; 11 University Medical Center, Theodor-Kutzer-Ufer 1-3, 68167 Mannheim, Germany; 12 Klinikum St. Marien, Mariahilfbergweg 7, 92224 Amberg, Germany; 13 Knappschaftskrankenhaus, Dept. of Neurology, Ruhr-University Bochum, In der Schornau 23, 44892 Bochum, Germany; 14 Ulm University Hospital, Albert-Einstein-Allee 23, 89081 Ulm, Germany; 15 University Hospital, Albert-Schweitzer-Campus 1, 48149 Münster, Germany; 16 Hospital Ost, Züricher Strasse 40, 28325 Bremen, Germany; 17 OnkoZert GmbH, Certification Institute of the German Cancer Society, Gartenstrasse 24, 89231 Neu-Ulm, Germany

**Keywords:** Multidisciplinarity, Certification, Quality of care, Quality indicators

## Abstract

**Background:**

Over the last decades numerous initiatives have been set up that aim at translating the best available medical knowledge and treatment into clinical practice. The inherent complexity of the programs and discrepancies in the terminology used make it difficult to appreciate each of them distinctly and compare their specific strengths and weaknesses. To allow comparison and stimulate dialogue between different programs, we in this paper provide an overview of the German Cancer Society certification program for multidisciplinary cancer centers that was established in 2003.

**Main body:**

In the early 2000s the German Cancer Society assessed the available information on quality of cancer care in Germany and concluded that there was a definite need for a comprehensive, transparent and evidence-based system of quality assessment and control. This prompted the development and implementation of a voluntary cancer center certification program that was promoted by scientific societies, health-care providers, and patient advocacy groups and based on guidelines of the highest quality level (S3). The certification system structures the entire process of care from prevention to screening and multidisciplinary treatment of cancer and places multidisciplinary teams at the heart of this program. Within each network of providers, the quality of care is documented using tumor-specific quality indicators. The system started with breast cancer centers in 2003 and colorectal cancer centers in 2006. In 2017, certification systems are established for the majority of cancers. Here we describe the rationale behind the certification program, its history, the development of the certification requirements, the process of data collection, and the certification process as an example for the successful implementation of a voluntary but powerful system to ensure and improve quality of cancer care.

**Conclusion:**

Since 2003, over 1 million patients had their primary tumors treated in a certified center. There are now over 1200 sites for different tumor entities in four countries that have been certified in accordance with the program and transparently report their results from multidisciplinary treatment for a substantial proportion of cancers. This led to a fundamental change in the structure of cancer care in Germany and neighboring countries within one decade.

## Background

In addition to breakthroughs that have been made in the diagnosis and treatment of cancers, considerable effort over the last 20 years has been put into ensuring that the state of the art of care is translated into everyday care for the entire population in need. Indeed, this issue is at the heart of the debate about the quality of cancer care and how to improve it [1–5]. Numerous campaigns have been initiated that aim at translating the best available medical knowledge and treatment into clinical practice. They typically define standards for how to measure, compare, and improve the quality of care and how to best incorporate multidisciplinarity [6–12].

Some initiatives have also developed certification and accreditation programs for quality assurance and quality development using rigorous data collection processes, quality indicators, on-site auditing and peer review. These campaigns are partly government-driven, but most have been initiated by professional associations [12–16]. The inherent complexity of the programs and discrepancies in the terminology used make it difficult to appreciate each of them distinctly and compare their specific strengths and weaknesses. To allow comparison and stimulate dialogue between different programs, we describe here the certification system organized by the German Cancer Society (*Deutsche Krebsgesellschaft*, DKG). We explain the rationale for the certification program, the development of the certification requirements, the process of data collection, and the certification process.

## Discussion

### Rationale and brief history of the program

In the early 2000s, analyses of the limited data available suggested that despite enormous financial resources the results regarding cancer survival in Germany were only moderate compared to the results obtained in other European countries [17]. This resulted in several initiatives aimed at improving care, such as the development of clinical guidelines of the highest quality level for the most frequent cancer entities and a novel Cancer Center Certification Program by the DKG. This program was initially introduced for breast cancer following the example of the EUSOMA effort (European Society of Breast Cancer Specialists, [14, 18]) and soon afterwards also for other types of cancer. The program was intended to put evidence-based guideline recommendations into practice in everyday care and to place multidisciplinary teams at the heart of the cancer care process. To ensure that evidence-based standards are met in the certified units an auditing system based on quality metrics was implemented. The Cancer Center Certification Program was originally initiated, and has since been continually developed, by the professional associations involved in cancer care, working groups, and patient advocacy groups. Participation in the certification system is voluntary. This means that there is no official (government) body that requests certification in order to be able to provide a particular cancer service, and the government is not involved in defining the criteria for certification.

**Fig. 1:**
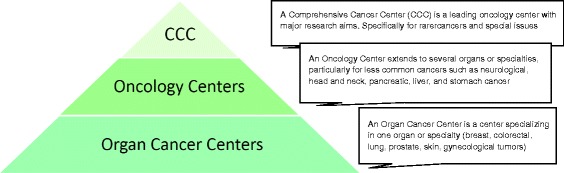
The three-tier model of certified cancer centers

The centers established within the DKG certification system are *tumor-specific networks* (for an overview of the various types of center, see Fig. 1) in which patients are treated in a comprehensive, interdisciplinary, and multi-professional manner. Disciplines that are essential for the treatment of a particular cancer are specifically defined for each type of center. In breast cancer centers for example these teams comprise gynecologists, radiologists, pathologists, medical oncologists, radiotherapists, and specialists in nuclear medicine (termed “main cooperation partners”). In addition, written agreements for on request services have to be made with representatives of the following fields: oncological nursing, psycho-oncology, hospice/palliative medicine, social services, patient advocacy groups, genetic counseling, physiotherapy, laboratory medicine, and supplier of medical products [19].

### Development of the DKG cancer centers in Germany

Shortly after the publication of the first Eurocare results [17], the DKG and the German Society for Breast Diseases (*Deutsche Gesellschaft für Senologie*, DGS) established the first set of criteria for certified cancer centers in accordance with the national clinical guidelines for breast cancer [20]. Subsequently, colorectal cancer centers (2006), skin cancer centers, gynecological cancer centers, lung cancer centers, and prostate cancer centers (all in 2008) were added to the certification program, each following guidelines of the highest quality level for the respective cancer entity. Each program has overlapping, general standards but also contains a set of distinct, cancer-specific requirements. Some of these requirements are translated into quality metrics that allow for comparisons across the different sites all over the country. The centers cover the whole spectrum of care for the respective tumor including prevention, early detection, treatment of the primary and advanced tumors, supportive and palliative care as well as psychooncology, social support and self-help groups.

The DKG program operates as part of the German National Cancer Plan led by the German Ministry of Health [21], that laid down a three-tier model of cancer centers in Germany consisting of Organ Cancer Centers (C), Oncology Centers (CC), and Comprehensive Cancer Centers (CCC, funded by the German Cancer Aid) (Fig. 1) [22]. Patient care must meet the same quality requirements in all aspects irrespectively of the institution and of its position in the three-tier model of the National Cancer Certification Program [23].

**Fig. 2:**
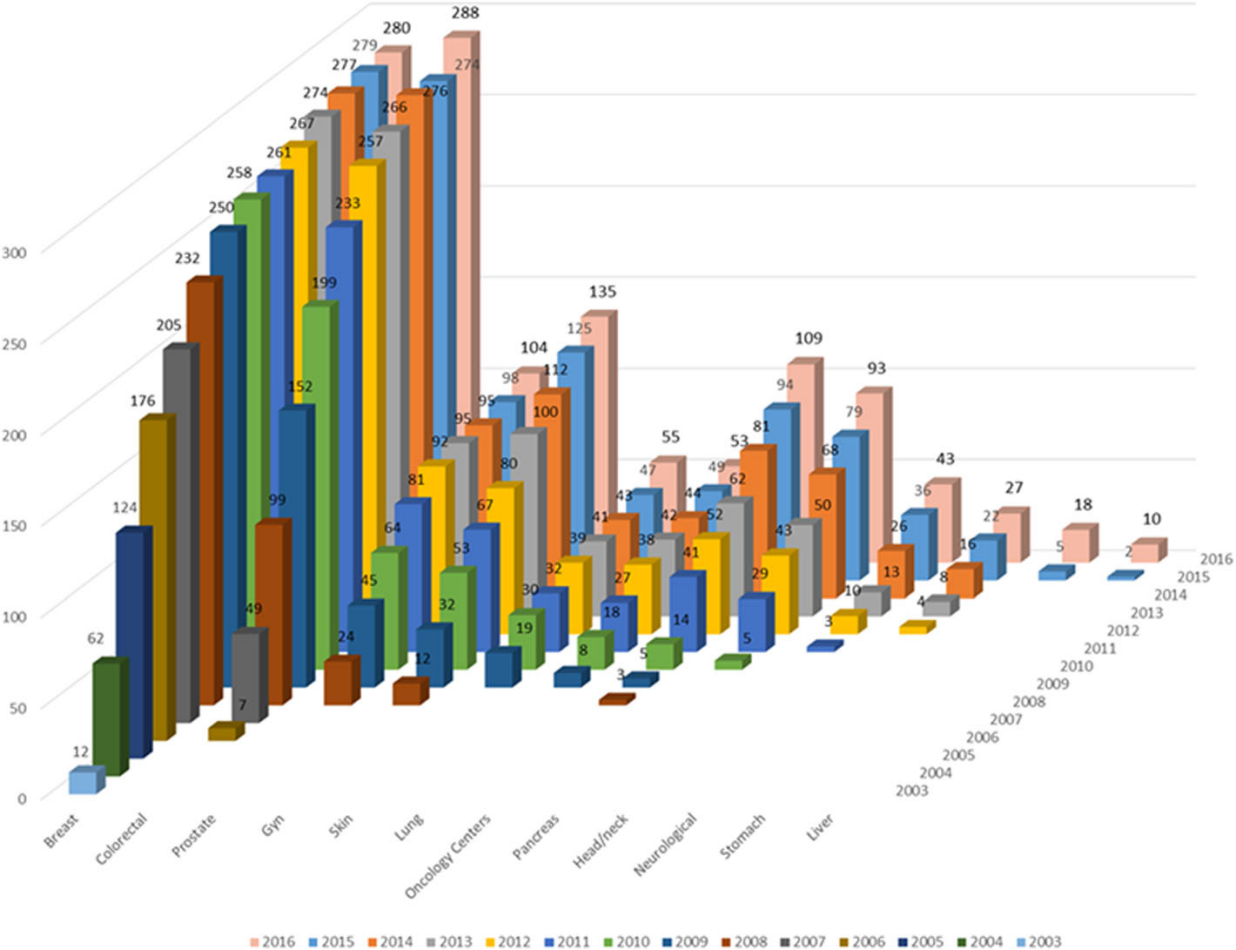
Numbers of center sites, 2003–2016

**Fig. 3:**
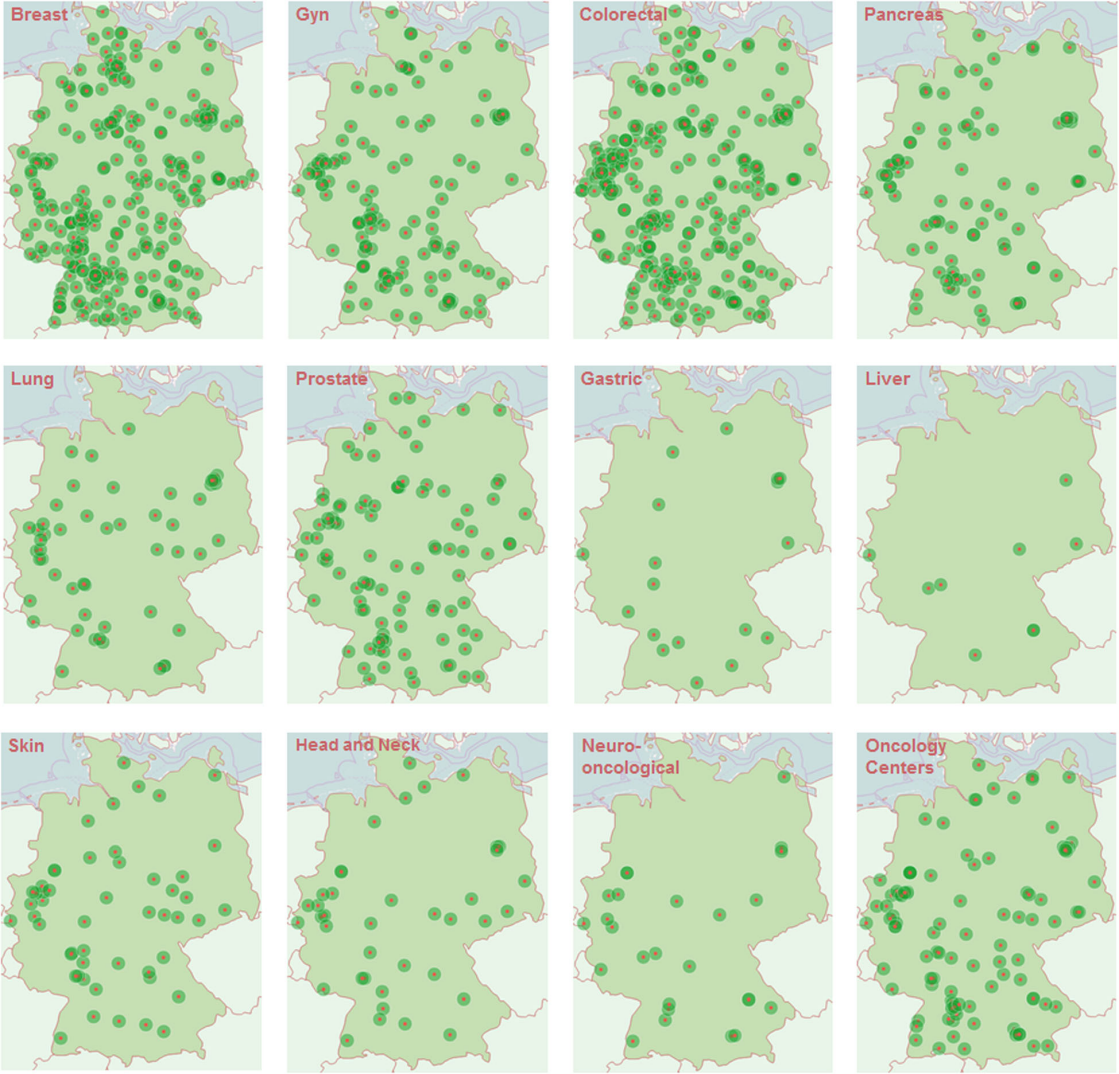
The distribution of centers in Germany

As of January 2017, there are 1200 certified Organ Cancer Centers, 109 Oncology Centers, and 15 Comprehensive Cancer Centers in Germany. Fig 2 demonstrates that this still entirely voluntary program is continuously growing. By now, approximately 40% of the population in Germany with primary cancers for which centers have been established are treated in certified institutions (Table 1). The geographic distribution of centers is illustrated in Fig. 3. Sixty sites are currently located in countries neighboring Germany — Italy, Switzerland, and Austria.

**Table 1:** Current status of the certification system

	Organ cancer centers							Modules				Oncology centers	Comprehensive cancer centers
	Breast	Colorectal	Gynecological	Skin	Lung	Prostate		Head/neck tumors	CNS tumors	Pancreas			
Current first certification	4	7	6	5	5	7		6	8	5		6	
Certified center	230	280	133	55	45	103		41	26	91		97	
Certified center sites	280	288	135	55	53	104		43	27	93		109	15
Primary cases, 2015	54,230	26,660	12,306	11,209^b^	17,731	19,932		6273^c^	5456^d^	4070		35,670	
New cases of cancer^a^	70,170	62,230	26,140	20,820 ^b^	52,520	63,710		15,628^c^	–	16,730		–	
Total share^e^	74.6%	41.4%	43.6%	50.8%	32.9%	29.6%		37.5%	–	22.9%		–	
Center sites abroad	11	10	9	4	2	7		4	1	6		6	

CNS, central nervous system

^a^Based on German registry data from 2012

^b^Only includes malignant melanoma

^c^New cases of head and neck tumor: mouth and pharynx ICD-10 C00–C06, C09–C14, larynx C32

^d^Neuroendocrine tumors (C70–C75, in the International Classification of Diseases for Oncology, ICD-O)

^e^Numerator: primary patients from centers in Germany, denominator: primary cases in Germany

As of: 31 December 2016, does not include data on the two most recently added modules (liver, stomach)

### Certification: Structure, standards, and fulfillment

**Fig. 4:**
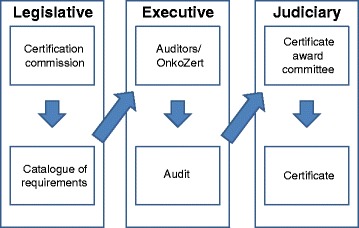
The separation of powers in the certification system

To provide the greatest possible degree of professional independence for those who develop and review the requirements and their implementation, and to avoid individual conflicts of interest, the certification system is divided into three branches that work independently of each other (Fig. 4 “Separation of Powers”).

1. The cancer type–specific Certification Committee (corresponding to a “legislative” body): This committee defines and develops the requirements specification for the certification of the respective centers based on evidence-based clinical guidelines of the highest quality (S3 level, with S standing for “Stufenklassifikation” or “step classification”) and supplemented by recent research results that are not yet part of the guidelines or refer to structural requirements e.g., staffing or technical infrastructure. The clinical guideline categories as defined by the German Association of the Scientific Medical Societies (AWMF) range from S1 to S3, with S1 being based on expert consensus only and S3 (the highest level) being evidence based, i.e. based on a systematic literature review following defined criteria; having a representative guideline panel including patients; and using a formal consensus finding process. Based on the core recommendations of the guidelines, quality indicators are derived and implemented into the certification requirements [24]. Recent research results and structural requirements, unless suggested by clinical guidelines, are based on expert consensus in the respective Certification Committee, allowing for example patients to suggest requirements that are then discussed. Each cancer–specific Certification Committee consists of 30–40 experts of all specialties and professional groups involved that have been delegated by their respective professional medical society, by scientific societies, working groups, and self-help organizations. The Certification Committees convene annually or biennially to discuss the results from the certified centers and ensure timely updates of the requirements specification.
2. Audit and data evaluation (corresponding to an “executive” body): To obtain the certificate for a specific cancer center the applicants must comply with the standards set out in the requirements specification developed by the relevant Certification Committee of the DKG. Data on the requirements are documented by each center using an electronic reporting system. Data are then reported to an independent certification institute, OnkoZert, where the documentation is formally checked for completeness and plausibility. To assure independence, OnkoZert manages the documents, organizes the audits and commissions the auditors but cannot influence the decision of awarding the certificate. Specially trained oncological medical experts from various specialties including surgery, pathology, gastroenterology, medical oncology, gynecology (i.e., the auditors) verify the implementation of the requirements and the validity of the documentation. Verification of the implementation of the requirements is done during an on-site audit of the documentation, of structures and processes at the centers. During these audits, the auditors examine actual patient files and discuss the results with the centers at the end of the site visit. The auditors prepare a written report about the data and the on-site visit. If any non-compliance with the certification requirements is observed during the audit, the auditor pronounces a so-called “deviation”. The center then has three months to remedy this deviation starting from the audit day. Positive remedying is the precondition for the award of the certificate. The auditors include a recommendation about the award of a certificate in their report. If the center fails to remedy a deviation, the auditor will recommend to not issue the certificate. Auditors must not be members of the Certification Committee.
3. Certificate Award Committee (corresponding to "jurisdiction"): The final decision whether to award or deny a certificate is taken by another independent body, the Certificate Award Committee. This committee consists of three medical experts who are not involved in the corresponding audit. The Committee awards or denies the certificates based on the audit report and the auditors’ recommendations. In 2016, 117 new certificates have been awarded and 14 certificates have been withdrawn because of not sufficient audit results.

### Data reporting

All data from the tumor documentation of the respective centers are submitted electronically to the independent certification institute OnkoZert. Here, a software called OncoBox that can interact with and extract data from the tumor documentation softwares used in Germany, runs a plausibility check of the data and calculates the quality indicators. Discrepancies are fed back to the centers for clearance. Cancer type and also center–specific results regarding the quality indicators are presented to the public in annual reports (e.g., [25]) and in scientific journals (e.g., [20, 26, 27]). Some of the requirements and quality indicators are the same for all center types and tumor entities, such as a study participation rate of 5% of patients, delivery of social services and psycho-oncological counseling to all patients, and discussion of all patients in specific multidisciplinary boards. However, most of the requirements are tailored to the specific center/tumor. As an example, one tumor-specific quality indicator for each center type is listed in Table 2. The quality indicators presented in the Table are derived from the clinical guidelines, unless otherwise indicated, and are part of the annual reports.

**Table 2:** Example quality indicators for each center/module type

Center/module	Quality indicator definition	Target value	Sites meeting the target in 2015	Median	Source
Breast	Pretreatment histological confirmation (numerator: patients with pretreatment histological diagnosis confirmation by means of a punch or vacuum biopsy; population: patients with initial procedure and histology showing invasive breast cancer or DCIS as primary disease)	≥ 90%	97.5% (268/275)	97.6%	Annual Report of Certified Breast Cancer Centers 2016 —Quality Indicators (in English) doi:10.13140/RG.2.1.1805.7204
Colorectal	Quality of the TME rectum specimen (information from pathology; numerator: patients with good to moderate quality TME [grade 1: mesorectal fascia or grade 2: intramesorectal excisions]; population: patients with radically operated rectal cancer)	≥ 70%	98.5% (257/261)	95.2%	Annual Report of Certified Colorectal Cancer Centers 2016 — Quality Indicators (in English) doi:10.13140/RG.2.1.3771.8001
Prostate	Percutaneous radiotherapy with hormone ablation therapy for locally confined prostate carcinoma with high risk (numerator: primary cases with additional neoadjuvant and/or adjuvant hormone ablation therapy; population: primary cases with prostate carcinoma T1–2 N0 M0 with high risk (PSA > 20 ng/mL or Gleason score ≥ 8 or clinical stage T2c) and percutaneous radiotherapy	No target in 2015	–	71.43% (93 sites)	Annual Report of Certified Prostate Cancer Centers 2016 — Quality Indicators (in English) doi:10.13140/RG.2.1.3673.4969
Lung	Combined chemoradiotherapy in stage IIIA4/IIIB (numerator: combined chemoradiotherapy in NSCLC primary cases in stage IIIA4/IIIB with ECOG 0/1; population: NSCLC primary cases in stage IIIA4/IIIB	No target in 2015	–	39.6%	Annual Report of Certified Lung Cancer Centers 2016 — Quality Indicators (in German) doi:10.13140/RG.2.1.3998.6327
Skin	Malignant melanoma: sentinel-node biopsy (SNB, numerator: primary cases in which SNB was carried out; population: primary cases of primary cutaneous melanoma with a tumor thickness of ≥1 mm and no evidence of locoregional or distant metastasis)	≥ 80%	72.7% (32/44)	85.2%	Annual Report of Certified Skin Cancer Centers 2016 — Quality Indicators (in German) doi:10.13140/RG.2.1.2227.2408
Ovary	Postoperative chemotherapy for advanced ovarian carcinoma (numerator: primary surgical cases of FIGO IIB–IV ovarian carcinoma with postoperative chemotherapy; population: primary surgical cases of FIGO IIB–IV ovarian carcinoma and chemotherapy)	No target in 2015	–	96.4%	Annual Report of Certified Gynecological Cancer Centers 2016 — Quality Indicators (in German) doi:10.13140/RG.2.1.1052.0568
Cervix	Histological confirmation	No target in 2015	–	100.0%	Annual Report of Certified Gynecological Cancer Centers 2016 — Quality Indicators (in German) doi:10.13140/RG.2.1.1052.0568
Head–neck	Imaging in patients with oral cavity carcinoma to determine N category (numerator: patients with CT or MRI examinations of the region from the cranial base to the superior thoracic aperture to determine the N category; population: primary cases of patients with oral cavity carcinoma)	No target in 2015	–	91.7%	Annual Report of Certified Head and Neck Tumor Centers 2016 — Quality Indicators (in German) doi:10.13140/RG.2.1.2788.7609
CNS tumors	Interdisciplinary case discussions (numerator: primary cases [elective patients: pre-interventional, emergency patients: post-interventional] presented at the tumor conference; population: primary cases), indicator not derived from clinical guidelines	≥ 95%	68.4% (13/19)	96.4%	Annual Report of Certified Neuro-Oncological Tumor Centers 2016 — Quality Indicators (in German) doi:10.13140/RG.2.1.2788.7609
Pancreas	Lymph-node examination (numerator: primary surgical cases of pancreas with ≥10 regional lymph nodes in the surgical specimen after completion of surgical treatment; population: primary surgical cases in pancreas (OPS 5–524 ff., 5–525 ff. only with ICD-10 C25) who have undergone lymphadenectomy)	No target value in 2015	–	90.0%	Annual Report of Certified Neuro-Oncological Tumor Centers 2016 — Quality Indicators (in German) doi:10.13140/RG.2.1.2788.7609

CT, computed tomography; DCIS, ductal carcinoma in situ; DOI, digital object identifier; FIGO, Fédération Internationale de Gynécologie et d’Obstetrique; ICD, International Statistical Classification of Diseases and Health-Related Problems, 10th Revision; MRI, magnetic resonance imaging; NSCLC, non–small cell lung cancer; PSA, prostate-specific antigen; TME, total mesorectal excision

With the number of DKG certified centers outside Germany constantly increasing, the German Cancer Society’s certification system has now become the largest in Europe. This provides many opportunities, including the ability to compare the quality of care not only within different regions of one country, but also across countries. Smaller countries with only a few centers, which would otherwise lack an appropriate comparator, can match their results with many centers from different countries and evaluate their development over time.

### Acquiring the certificate: The center perspective

The certification criteria are outlined on the website of the independent certification institute OnkoZert (www.onkozert.de). Once a center has decided to apply for its initial certificate, it submits an inquiry form 3 to 6 months prior to the projected audit date. The inquiry form is reviewed by OnkoZert and a judgement is made on whether the center is in principle suitable for certification or whether its current structures make certification impossible. It is recommended that the centers then submit the necessary data 2 months prior to the audit date. The documents are formally reviewed and passed on to the responsible auditor(s), who provide(s) a written assessment about the contents. This assessment is then sent to the centers so that they can use it to make final adjustments regarding structures and processes. The subsequent on-site audit takes one or two days, depending on the type of audit, and includes auditing of all departments that shall participate in the certified center as well as of external cooperation partners. Relevant documents such as patient charts are reviewed on site and compared with the data submitted. Discrepancies between the actual situation and the certification criteria must be corrected within 3 months after the audit and are then re-evaluated by the auditor on paper or on site, depending on the specific issue. The auditor submits the audit report to OnkoZert, which verifies the data, and passes the audit report on to the Certificate Award Committee. Finally, the latter decides on denying or awarding of the certificate, which is then valid for 3 years. Full reevaluation-audits take place every 3 years, and surveillance audits — i.e., shorter audits focusing on the results for key figures and quality indicators are performed every year.

## Conclusions

The DKG Cancer Center Certification program is a unique program: It is entirely voluntary, covers the most relevant tumor entities in a steadily increasing proportion, is based on guidelines of the highest quality, is run by experts in the field independent of governmental influence and comprises the whole spectrum of cancer care from prevention to screening, multidisciplinary treatment, social and psychooncological, supportive and palliative care. By implementing guidelines of the highest quality and the best available medical knowledge into everyday care it aims at comprehensively improving the quality of cancer care across all relevant tumor entities and across all regions. An important goal of the program is to also make quality of care transparent across all sites and thereby allowing to compare the centers based on objective indicators. This shall in turn stimulate mutual learning among the participating certified units.

The certification program has led to several paradigm shifts in Germany. Only three shall be outlined here. First and foremost, multidisciplinarity in cancer care has become widely established and is not limited to the multidisciplinary tumor boards. This is a development that would not have been foreseeable 15 years ago. The way in which the individual patient is treated no longer depends on one clinician alone, but includes expertise from the relevant providers within the network, who are specified by name. This multidisciplinary approach is not limited to medical specialties, but also includes psycho-social care [28] as well as nursing and palliative care, to which every patient is entitled. Secondly, the “separation of powers” in the certification system facilitates fair processes for the development and evaluation of requirements and for awarding certificates. Specific professional groups cannot play a dominant role. Thirdly, the collection and reporting of quality-of-care data that are implemented throughout the certification program [27], makes provider evaluation indicator-based and allows to compare the results and enables mutual learning. Indeed, due to the success of this program, comparisons are not limited to one country, but also include centers in other countries including Switzerland, Austria, and Italy. This provides an opportunity to make the quality of cancer care comparable on a European scale and to expand collaborations on this topic between scientific societies in different countries.

## Abbreviations

C: Organ Cancer Center
CC: Oncology Center
CCC: Comprehensive Cancer Center
DGS: Deutsche Gesellschaft für Senologie (German Society for Breast Diseases)
DKG: Deutsche Krebsgesellschaft (German Cancer Society)
EUSOMA: European Society of Breast Cancer Specialists
SNB: Sentinel-node biopsy
